# Supplementary material for: The Promising Potential of Triploidy in Date Palm (Phoenix dactylifera L.) Breeding
Source: Plants (Basel). 2024 Mar 12;13(6):815. doi: 10.3390/plants13060815 (PMC10975707; doi:10.3390/plants13060815)
Supplement: Supplementary file 1 [file plants-13-00815-s001.zip › plants-2843222-supplementary.pdf]

Supplementary material

Table S1. Mean values of selected morphological variables of the diploid ('Deglet Nour': T0) and triploid genotypes (T1, T2, T3 and T4) Lp: Length of the palm; Tr: Thickness of the rachis; Wp: Width of the palm at the base of the petiole; Nsp: Number of spines; Tsp: Max. thickness of the spines; Lsp: Maximum length of spines; NLf: Number of leaflets; Wsp: Maximum width of spines in midleaf; Lsm: Max. length of spines in the middle of the palm; Las: Length of the apical spine; Was: Width of the apical spine. Values followed by different letters denote statistically significant differences (Student–Newman–Keuls (S-N-K), $p \leq 0.05$).

Crown	Cultivar	Lp	Tr	Wp	Nsp	Tsp	Lsp	Nlf	Wsp	Lsm	Las	Was
	T0	2.54±0.18c	2.67±0.20b	9.91±0.43bc	34.22±3.27a	0.751±0.03b	21.05±1.51b	92.88±7.91d	2.552±0.05b	39.93±0.75d	26.94±1.04d	2.02±0.04c
	T1	2.39±0.08c	3.51±0.46a	9.144±1.27c	14.22±1.48c	0.477±0.06c	26.60±1.79a	74.11±9.86f	3.255±0.58a	56.41±1.55a	35.30±2.38b	1.95±0.38c
All	T2	2.74±0.07b	3.43±0.61a	11.04±0.35b	35.22±1.09a	0.58±0.17c	15.36±2.33d	104.30±6.68c	2.822±0.38b	52.48±0.60b	32.92±3.20bc	2.80±0.77b
	T3	2.95±0.30a	3.16±0.08a	9.614±1.16c	27.22±3.11b	0.577±0.04c	20.56±0.52b	132.6±5.85a	2.78±0.22b	46.93±3.70c	31.60±3.73c	1.70±0.52c
	T4	3.05±0.10a	3.50±0.13a	10.90±1.54b	27.88±1.69b	0.588±0.07c	21.04±0.14b	125.2±6.77b	2.82±0.36b	53.38±6.53ab	34.57±2.09b	2.66±0.18b
	T0	2.59±0.01c	2.62±0.03d	10.26±0.30d	38.00±1.00a	0.786±0.00b	20.76±0.32b	85.00±1.00d	2.56±0.05c	40.10±0.10f	27.56±0.05d	2.03±0.05d
	T1	2.34±0.00d	4.08±0.17a	10.80±0.26c	16.00±1.00e	0.466±0.05d	25.66±3.21a	65.00±2.00f	3.63±0.11a	56.83±1.60a	36.33±1.15b	2.446±0.10c
Base	T2	2.78±0.03b	4.23±0.20a	10.90±0.09c	35.66±1.52b	0.806±0.02b	14.73±2.66c	97.66±2.51c	2.93±0.05b	52.63±0.77b	36.83±0.37b	3.46±0.13b
	T3	3.06±0.05a	3.23±0.05c	11.10±0.09c	28.66±1.15c	0.533±0.05d	20.66±0.41b	125.60±1.15a	2.96±0.05b	51.16±0.25c	36.46±0.55b	2.40±0.03c
	T4	3.00±0.09a	3.59±0.03b	12.90±0.26b	26.66±0.57d	0.666±0.05c	21.00±0.19b	118.00±1.00b	2.96±0.05b	45.00±0.60e	33.93±0.70c	2.48±0.02c
	T0	2.71±0.06b	2.46±0.05c	10.03±0.25c	34.00±1.00a	0.76±0.01b	22.90±0.26b	91.00±1.00d	2.50±0.00d	39.10±0.10e	25.56±0.25e	2.00±0.00c
	T1	2.33±0.01c	3.03±0.05b	8.13±0.30e	13.33±0.57d	0.533±0.05cd	27.36±0.55a	70.66±2.08f	3.63±0.20a	57.60±0.52a	32.33±0.66c	1.60±0.1d
Medium	T2	2.78±0.03b	2.96±0.05b	10.93±0.51b	35.00±1.00a	0.50±0.00d	17.8±0.19d	103.3±3.51c	3.20±0.09c	52.33±0.75c	29.56±0.55d	3.16±0.15a
	T3	3.22±0.06a	3.11±0.10b	9.23±0.25d	29.66±0.57b	0.596±0.01c	21.03±0.25c	133.3±0.57a	2.496±0.04d	46.96±0.15d	29.56±0.60d	1.30±0.01e
	T4	3.15±0.05a	3.56±0.05a	10.27±0.14bc	30.00±0.00b	0.593±0.03c	21.03±0.05c	124.6±0.57b	3.14±0.05c	55.86±1.62b	37.16±0.76b	2.64±0.07b
	T0	2.33±0.15c	2.93±0.05bc	9.433±0.20c	30.66±0.57b	0.706±0.01b	19.5±0.36c	102.6±2.51d	2.59±0.01c	40.60±0.69f	27.70±0.09c	2.03±0.05c
	T1	2.5±0.02bc	2.43±0.05a	8.50±0.26d	13.33±0.57e	0.433±0.05d	26.76±0.25a	86.66±1.52e	2.50±0.10c	54.80±0.72b	37.23±0.77b	1.82±0.06c
Upper	T2	2.65±0.04b	3.10±0.10b	11.30±0.26b	35.00±1.00a	0.433±0.05d	13.56±0.51e	112.00±2.00c	2.33±0.05d	52.50±0.45c	32.36±0.51b	1.79±0.01c
	T3	2.57±0.11b	3.15±0.05b	8.51±0.08d	23.33±1.52d	0.603±0.00c	20.00±0.19c	139.00±1.00a	2.87±0.05b	42.66±0.75e	28.76±1.15c	1.39±0.01d
	T4	3.00±0.10a	3.29±0.04b	9.526±0.12c	27.00±1.00c	0.506±0.02d	21.1±0.17b	133.00±3.60b	2.34±0.05d	59.30±0.75a	32.63±0.35b	2.86±0.15b
